# Head and Neck Cancer Care Experiences of First Nations Australians: Insights From Patients and Carers

**DOI:** 10.1111/ajr.70238

**Published:** 2026-08-09

**Authors:** Stephanie Ng, Tamara Butler, Rebecca Packer, Joanne Tesiram, Lynette Koina, Kalisha Soe, Elizabeth C. Ward, Gail Garvey, Bena Brown

**Affiliations:** ^1^ School of Health and Rehabilitation Sciences The University of Queensland Brisbane Queensland Australia; ^2^ Centre for Functioning and Health Research Metro South Hospital and Health Service Brisbane Queensland Australia; ^3^ Princess Alexandra Hospital, Metro South Hospital and Health Service Brisbane Queensland Australia; ^4^ Yardhura Walani, National Centre for Aboriginal and Torres Strait Islander Wellbeing Research College of Law, Governance and Policy Australian National University Canberra Australian Capital Territory Australia; ^5^ Southern Queensland Centre of Excellence in Aboriginal and Torres Strait Islander Primary Healthcare Metro South Hospital and Health Service Brisbane Queensland Australia; ^6^ First Nations Cancer and Wellbeing Program, School of Public Health The University of Queensland Brisbane Queensland Australia

**Keywords:** care experiences, First Nations Australians, head and neck cancer, Yarning

## Abstract

**Objective:**

There is a paucity of evidence exploring the experiences and care needs for First Nations Australians with head and neck cancer (HNC). The aim of the study was to explore the journey of HNC diagnosis, treatment, and early recovery for patients and carers to inform service improvement.

**Setting, Participants and Design:**

First Nations HNC patient and carer (*n* = 19) experiences were explored qualitatively through Yarning. Data was analysed using reflexive thematic analysis, with First Nations researcher involvement embedded in all stages of data collection and analysis.

**Results:**

Key themes identified included: Strengths and protective factors; Coordination of care; Quality and continuity of care; Cultural safety; Communication and information; Cultural connectedness in hospital; and Gaps in health system support. The Strengths and protective factors theme was noted to be interwoven throughout the other themes and supported participants as a safety net during their care. Practical recommendations to the HNC care pathway were also offered under each theme.

**Conclusions:**

The seven themes identified provided insights into the HNC care experiences for First Nations Australians. These findings and participant recommendations will inform a co‐design process to create an optimised care pathway.

## Key Points

**What is already known on this subject?:**

Whilst there is growing literature around First Nations Australians and their experiences with the cancer care pathway more generally, little is known about the experiences specifically within head and neck cancer care, resulting in limited first‐hand evidence to guide service improvement.

**What does this study add?:**

The study enabled understanding of First Nations Australians and their experiences through the current head and neck cancer care pathway, using a strengths‐based and decolonising approach. The findings, which include participant‐generated recommendations, will be utilised in a co‐design process to inform changes for a more optimised head and neck cancer care pathway for First Nations Australians.

## 1 Introduction

Head and neck cancer (HNC) is the sixth most common cancer worldwide [1] and poses unique challenges to those affected given its frequent impacts on core functions such as breathing, swallowing, and communication [2]. HNC survivors often experience significant functional, financial, and psychosocial impacts associated with the disease and treatment sequelae, both immediate and in the longer term [1]. Whilst HNC incidence, mortality rates, and overall survival outcomes have improved over recent decades in Australia [3], this is not reflected equally across all populations. Aboriginal and/or Torres Strait Islander people, hereafter respectfully referred to as First Nations Australians, are one such cohort continuing to experience disproportionate impacts of the disease. The legacy of colonialism and oppressive government policies, which included dispossession and displacement, forced family separation, denial of rights, and disconnection from culture [4], has directly contributed to ongoing disadvantage and poor health outcomes [5]. Specifically within HNC, First Nations Australians continue to face significant disparities across incidence rates [6], treatment timing [7], treatment completion rates [8], and survival [9].

Beyond HNC specifically, the growing evidence around First Nations Australians and experiences in the cancer care system highlight a number of issues that deter service access, such as the financial and emotional impact of travel away for treatment [10], competing demands of kinship or cultural roles [11], inadequate cross‐cultural communication from healthcare staff [12], as well as past experiences of racism within the health system [13]. To address these challenges, the Optimal Care Pathway (OCP) for Aboriginal and/or Torres Strait Islander people provides key direction and principles for application along the cancer continuum [14], but covers all cancers more broadly and is intended to be used alongside the tumour‐specific HNC OCP developed for the wider population [15]. Therefore, despite this foundational pathway guidance, there remains no specific information on how to best deliver HNC services for First Nations Australians. Furthermore, little is known about the experiences of First Nations people with HNC across the continuum of care, resulting in limited first‐hand evidence to guide improvements to the cancer care pathway. Therefore, the aim of this study was to understand the journey of HNC diagnosis, treatment, and early recovery for First Nations people and their carers in Queensland.

## 2 Materials and Methods

### 2.1 Ethics Statement

This study was conducted in accordance with NHMRC Ethical Guidelines for Research with Aboriginal and/or Torres Strait Islander people and the Australian Institute of Aboriginal and Torres Strait Islander Studies (AIATSIS) Code of Ethics for Aboriginal and Torres Strait Islander Research. Specific approval for research with Aboriginal and Torres Strait Islander peoples was granted by the Metro South Health Human Research Ethics Committee.

### 2.2 Design

A qualitative descriptive approach was selected, primarily for its flexibility in understanding patient experiences and perceptions, and has been well‐utilised in nursing and other healthcare settings [16]. This approach is designed to be dynamic and inductive and reflect philosophical theories that understand reality is perceived differently depending on the subject, and therefore is best aligned with constructivism and an interpretative approach [16]. Despite this flexibility, a qualitative descriptive approach is still grounded in Western roots. Therefore, the research team intentionally employed decolonising and strengths‐based strategies to address this. Decolonising research practices recognise and shift from existing Western frameworks and rules to privilege First Nations ways of knowing, being, and doing [17] and a strengths‐based approach emphasises First Nations cultures and strengths as being foundational to any growth or change [18]. First Nations leadership, engagement, and reciprocity were prioritised from conceptualisation of the study, through data analysis and dissemination. Yarning [19], a culturally appropriate qualitative research technique, was employed for data collection, and reflexive thematic analysis [20] was used to describe patients and carers' experiences with HNC care. As such, First Nations participant voices were privileged and researcher subjectivity and influence were also emphasised throughout the yarns and analysis. These strategies aimed to ensure research conducted was accountable and useful, being of benefit to the community, and addressed the priorities identified by local First Nations leaders and the HNC survivor on the research team. In addition, this research collaboration enabled valuable two‐way capacity building for both First Nations and non‐First Nations research team members.

### 2.3 Positionality

The research team recognises the importance of reflexivity, whereby individuals reflect on how their experiences and background can influence the data [21], and how it serves as a critical component of both decolonising practice and qualitative research. S.N. is a non‐First Nations early career researcher and allied health clinician in HNC care. T.B. is an Aboriginal (Undumbi) public health researcher with a focus on improving cancer outcomes for Aboriginal and Torres Strait Islander people. R.P. is a non‐First Nations experienced researcher with expertise in qualitative methodologies and survivorship in HNC. J.T. is an Aboriginal (Wiradjuri) experienced Clinical Nurse Consultant and is a fierce advocate for Aboriginal and Torres Strait Islander peoples' equity. L.K. is an Aboriginal (Butchulla, Waanyi) researcher with a focus on improving Aboriginal health and wellbeing. K.S. is an Aboriginal (Kuku Yalanji/Kalkadoon) and Samoan researcher and health practitioner‐in‐training, who brings lived experience and professional expertise to research for Aboriginal and Torres Strait Islander peoples. E.W. is a non‐First Nations experienced researcher with long standing research interests in improving the care pathway for all people with HNC. G.G. is an Aboriginal (Kamilaroi) senior researcher with extensive experience within First Nations health research. B.B. is a non‐First Nations researcher and allied health clinician whose work focuses on equitable health service delivery and community‐centred research with Aboriginal and Torres Strait Islander people.

### 2.4 Setting/Context

The study was conducted in Queensland, Australia, which spans an area of 1 727 000 km^2^ [22], and has a population in excess of 5 million people [23]. As of 2021, First Nations people represented 4% of the total Queensland population, with the majority residing outside of the state capital city, Brisbane [24]. A free statewide public health system is available to all residents in Queensland via 16 distinct Hospital and Health Services (HHS), each responsible for delivering care in their respective geographical areas. Metro South HHS, where the current study was based, services the south side of Brisbane, and includes the lands of the Yugambeh, Quandamooka, Jaggera, Ugarapul, Turrbal, and Mununjali peoples. The Princess Alexandra Hospital (PAH), within Metro South HHS, is a tertiary referral centre for HNC patients from across the state, as well as interstate and from the nearby Pacific Islands.

### 2.5 Participants

**FIGURE 1:**
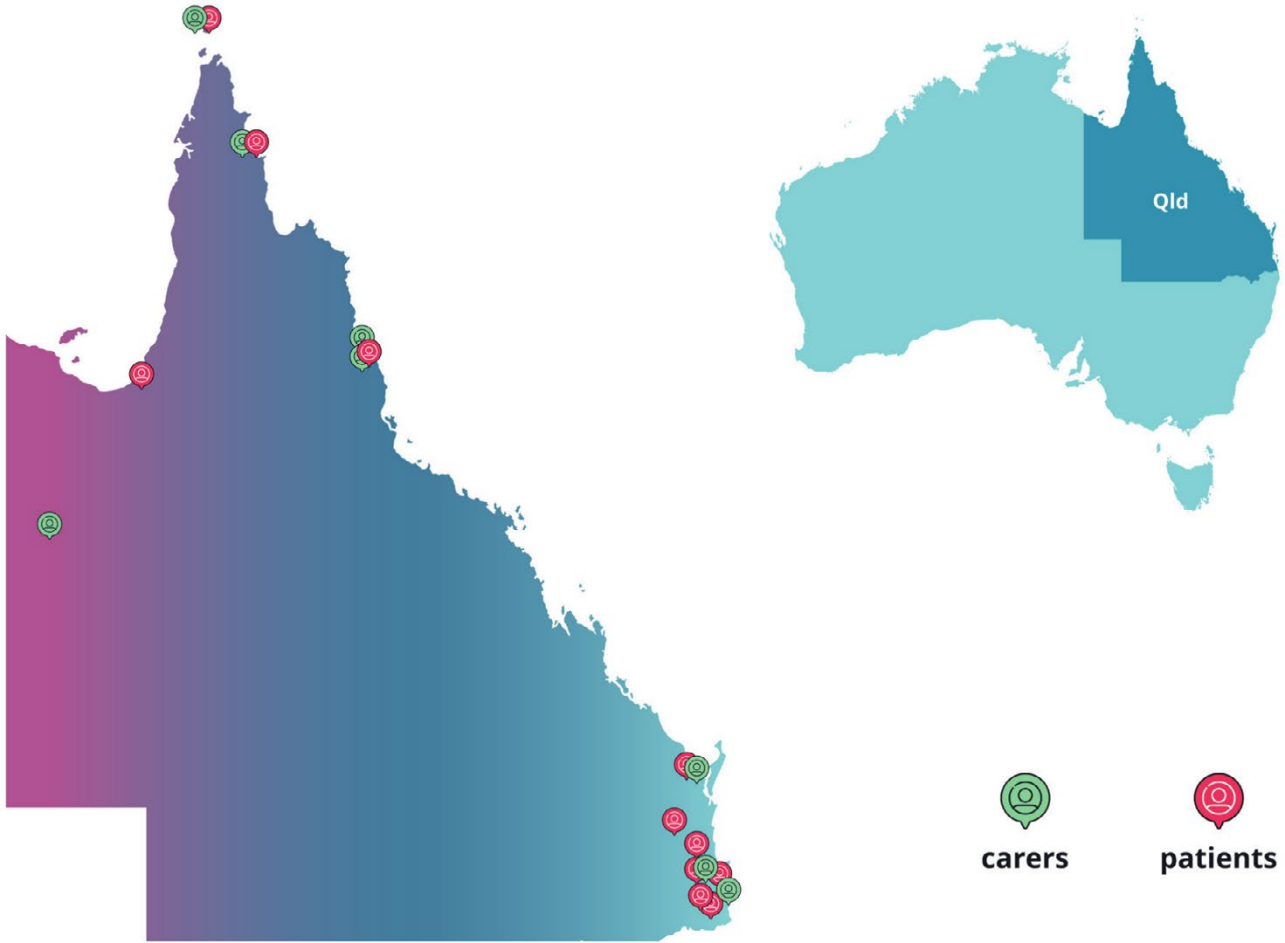
Participant geographical spread.

First Nations people aged over 18 years, diagnosed with HNC, and their carers were identified through the PAH multidisciplinary head and neck clinic lists (2022–2024) and were invited to participate in the study. Purposive recruitment was undertaken and ceased when all the eligible participants able to be contacted were reached. Consent was obtained either in written or verbal (recorded) form with a First Nations investigator present, and participants were advised consent could be withdrawn at any time without consequence. The final participant group included a diverse cohort of 8 carers and 11 patients with HNC (*n* = 19), mostly aged over 35, and were all geographically spread across Queensland with more than half (63%) from regional, rural, or very remote areas (Figure 1). Both Aboriginal and Torres Strait Islander people were represented in this cohort (Aboriginal *n* = 14, Aboriginal and/or Torres Strait Islander *n* = 4), with a reasonably even split of male (47%) and females (53%). Most participants or their families (68%) had received multimodality treatment and the majority (79%) were less than 2 years since treatment completion at the time of research participation.

### 2.6 Data Collection

Data collection was undertaken between June and November 2024. Yarning emphasises building of trust and relationships, and facilitates information gathering through a relaxed conversation style interaction [19]. The sessions were facilitated in person or via telephone by a First Nations investigator (J.T. or K.S.) together with the lead investigator (S.N.) to support cultural safety. All sessions were conducted in English, and commenced with a social yarn to establish relationality and trust, which then led into the research yarn, as described by Bessarab and Ng'andu [19]. A yarn guide was utilised to prompt reflections along the cancer care continuum, as well as identify any perceived facilitators and barriers to care. The interviews were recorded with consent and transcribed verbatim for reference, where possible. Participants with verbal communication challenges were offered other response options, for example, written response to questions, or supported through communication strategies with lead investigator (speech pathologist), such as drawing, gesturing, repeating and rephrasing to check messages. Where transcriptions were not possible due to severely reduced intelligibility (*n* = 2), a summary of the transcript was generated and cross checked with both researchers present to ensure the main messages were appropriately captured. All participants were offered a copy of transcripts or summaries and were invited to make any amendments.

### 2.7 Data Analysis

The transcripts were entered into Nvivo 14 software. Reflexive thematic analysis with an inductive, data‐driven approach was conducted as guided by the steps outlined by Braun and Clarke [20, 25]. Whilst not typical in this method, a codebook was used to facilitate team analysis to ensure appropriate consideration of First Nations worldviews. Phase 1: S.N., B.B., and L.K. separately familiarised themselves with the transcripts. Phase 2: This group then gathered to discuss the data and generated initial descriptors for the coded statements, which were later refined based on feedback received from T.B. and R.P. S.N. and L.K. subsequently reviewed and coded the remaining transcripts. S.N., T.B., L.K., and B.B. then reviewed the summarised table of codes, descriptors, and quotes to ensure general agreement. Phase 3: S.N. generated initial broad themes through identifying patterns of meaning with early feedback from the subgroup of researchers. Phase 4: First Nations researchers (T.B., G.G.) and HNC research expert (E.W.) then provided feedback to these themes to ensure participant perspectives were appropriately reflected. Phase 5: The final list of themes was reviewed by the full research team for agreement.

## 3 Results

**FIGURE 2:**
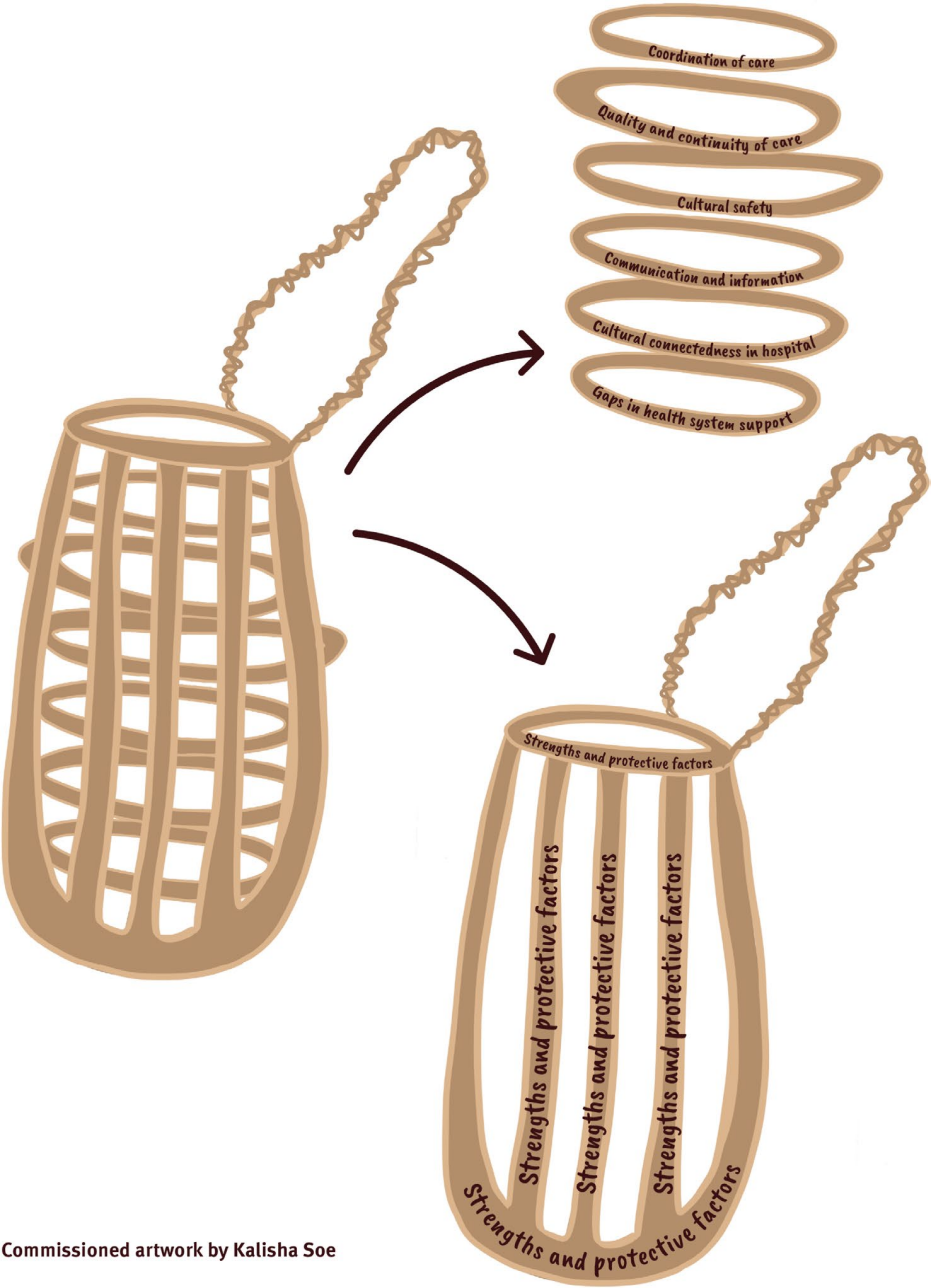
Visual representation of themes, with the Strengths and protective factors theme interwoven through the others.

Reflexive thematic analysis exploring the participants' experience of HNC care yielded seven main themes: (1) Strengths and protective factors; (2) Coordination of care; (3) Quality and continuity of care; (4) Cultural safety; (5) Communication and information; (6) Cultural connectedness in hospital; and (7) Gaps in health system support. The Strengths and protective factors theme was considered an integrative theme, with aspects woven throughout the other themes to support the challenges of navigating a complex cancer diagnosis, care experiences and health system (Figure 2). Quotes provided within the themes below have been identified by their participant number and as arising from a patient (P) or carer (C).

### 3.1 Strengths and Protective Factors: A Safety Net for Care

Strengths and protective factors were integrated throughout the stories shared by participants as a safety net against the challenges of the HNC care experiences and the health system. The extended kinship network of mutual support, cultural obligations to provide care, emphasis on protecting each other from burden, as well as a sense of a shared journey, were all integral in sustaining participants through the challenges of HNC care. ‘Yeah, I had to sort of take lead for the family and tell everyone… yeah it was sort of decided there that I would go through a process with her’ (C16). Furthermore, carers advocating and anticipating care needs for the patient were salient throughout the yarns, highlighting the strength of support offered, as well as at times a deep spiritual connection among family. ‘And yeah with her not being able to talk, I knew what she wanted, and I don't know how. I just know her.’ (C16). In addition, patient autonomy and self‐advocacy were also interwoven through the care experience, which pushed for the urgent interventions that they needed and prevented them falling through the gaps of the health system: ‘Lucky I asked to get referred to a specialist … he put the camera up my nose and he said “yeah it could be the big C”’ (P3).

This integrative theme is reflective of the collective nature of health and healing for many First Nations Australians, as well as the strength and determination of participants to navigate the HNC health system. It is clear that a more optimised care pathway is one that centres the kinship network, and prioritises the voices of those receiving care, thereby strengthening the weave of the existing safety net.

### 3.2 Coordination of Care: Flexible and Streamlined Care Is Key

This theme relates to care coordination across the HNC continuum experienced by patient and carer participants, and how it influenced the ease of accessing the health system. Many participants raised issues including those associated with coordination of appointments, between different health services, various health professionals (e.g., dentist and general practitioner), and community programs. Whilst most recalled seeing First Nations liaison officers, nurse navigators, or social workers and found them helpful for coordination support, these roles were not consistently available or offered through the care pathway. The resultant frustration and sense of disjointed care meant participants were more likely to slip through gaps in the care pathway. ‘He was getting angry with that [accessing multiple services] because he said “why can't these services work together? Why do I have to go here and here?”’ (C6). Furthermore, for those that needed to travel for care, taking on responsibilities of coordination even after contact with a coordinator role and the stress when these arrangements were inconvenient or unsuitable, were seen as an additional burden to the treatment journey. ‘They didn't book us for that long so I had to move my daughter twice… from one scary place, she rang me, had no lights in the hallway. I was lucky I was there at the one place because I don't think I would have stayed in that other place that was really dirty, and dark, scary for her’ (C12). On the other hand, positive care coordination experiences were captured, which included flexibility in appointment timing, modality options, e.g., telehealth, as well as early notification of appointments in order to organise transport and community support. These initiatives actively encouraged patient participation in care, tightening the mesh of the safety net and reducing the chance of slipping through the gaps.

The challenges associated with coordination within the health journey served as an additional barrier and deterrent to engaging with the HNC care system. However, the key facilitators offered in this theme may enable First Nations Australians to access and maintain connection with the care pathway moving forward.

### 3.3 Quality and Continuity of Care: More Expertise and Personalised Care Wanted

Quality and continuity of care was identified as a key theme in many yarns and was associated with the level of satisfaction for the care received. Participants expressed concerns around a misdiagnosis of initial symptoms, a perceived limited HNC or treatment specific knowledge, as well as the lack of consistency of local care team, all of which contributed to delays, uncertainty, and reduced confidence in the care received: ‘And I went to the doctor here [at home location] and they don't know. Couple weeks later I couldn't swallow so I went back to the doctor. Different doctor. Always different up here’ (P1). Notably, advocacy and persistence from patients and families were often crucial in ensuring they did not fall through the cracks. Furthermore, some participants were apprehensive of not receiving the highest quality of care, such as when being seen by specialists‐in‐training or student health professionals, not receiving the attention or support needed during a time of increased vulnerability and fear on the hospital ward, e.g., with a tracheostomy (altered airway requirements) or inability to speak, as well as a lack of personal touch in care interactions: ‘When we saw [radiation oncologist] it was like very quick, like there's a revolving door, doctors, you in for like two minutes and then you're out’ (C4).

This theme captured some participants' sentiments of uncertainty, angst, and feeling overlooked due to the care delivery at times, which may reduce trust and discourage access to the health system. Both patients and carers emphasised improvement of staff knowledge and expertise, and more personalised care, being crucial in instilling confidence in the care they receive.

### 3.4 Cultural Safety: ‘They Have to Learn Our Ways’ (C2)

Participant experiences of cultural safety and respect within the health system varied. One patient participant and her family were disappointed that their cultural ways regarding men's and women's business were not respected and hindered their participation in therapy and recovery process: ‘And the culture side of the cleaning, like bathing her, like our way up here, only women touch women. And I put a sign above her bed to say – “only women to do hygiene…” but they kept on doing it, sending men, and then she would just ring us, not talk, and we knew to get up to that hospital and there'd be a man bathing her’ (C12). This emphasised again the essential role of family as the safety net to promote what is needed for feeling safe, healing and wellbeing for First Nations people.

Some participants also indicated a lack of trust in the staff or hospital system, exacerbated by examples of errors or non‐routine collection in Indigeneity, resulting in increased caution and scepticism with all healthcare interactions: ‘I wouldn't really disrespect a nurse or a doctor because … I'm like, they could do anything. They can do anything bad, do you know what I mean? Because you can't trust anybody’ (C2).

The lack of trust and cultural safety shared by participants may confirm or worsen existing perceptions of a systemically racist and discriminatory healthcare system, which may create a further deterrent for accessing and engaging with care. Proposed improvements from participants, such as cultural education for staff and separate men's and women's areas in hospital, may help to meet the cultural needs of many First Nations people.

### 3.5 Cultural Connectedness in Hospital: ‘It Helps to Process. You Know You've Got Your Culture’ (P5)

Participants highlighted the need for connection to culture to endure the HNC journey and to promote holistic wellbeing. One participant reported whilst they appreciated opportunistically being able to attend cultural events during NAIDOC Week (National Aboriginal and Islander Day of Celebration) during their hospital admission, they would have valued having access to other activities on the ward: ‘Cultural activities, whether it's weaving or you know something like that would have been really beneficial, you know, like it's that creative side that you're not having to think too much’ (C16). Some participants felt having Elders from their community visit in hospital would ‘lift them up’ (C12) and help ‘keep you positive’ (P5). One participant also felt access to traditional healers and a smoking ceremony, particularly at the start of the journey, would have been helpful. Another patient participant shared an inability to access his usual bush medicine whilst in the tertiary metropolitan hospital. These examples represent missed opportunities to draw on culture for inner strength and wellbeing, which would in turn strengthen the fabric within the safety net woven throughout the journey. This theme captured participant suggestions for improved access to cultural activities and traditional healing, which may be key to supporting First Nations Australians to feel safe and connected in hospital and thereby encourage their ongoing engagement with essential HNC services.

### 3.6 Communication and Information: Needs to be Tailored

Communication and interactions with the healthcare staff played an important role in shaping the healthcare experience for First Nations Australians with HNC and their families. Most participants felt they could have benefitted from more information or explanation about the HNC journey, cancer itself, as well as HNC awareness in the community. Some carers also remarked they wanted further information on helping the patient through the cancer treatment or caring for the patient at home, highlighting the importance of supporting the safety net: ‘It's been rough because he could hardly eat, so, you know, you want to get them to eat because they need to build up their strength again. That part's hard. I don't know, just not a lot to come home with to say you know “do this, do that, try different things”’ (C4).

Some participants shared that some interactions with staff left much to be desired. These included instances of feeling they were not being listened to by healthcare staff or that staff would shy away from sensitive topics, such as prognosis, death, and dying, thereby hindering any further difficult discussions with the family. These sentiments emphasise the need for staff to take the time to invest in open and honest conversations: ‘Doctor [surgeon] actually came in and seen us, and… he was just very honest. And you know that bit there, I appreciated that he was there to explain to us what had happened in the steps and that things did change once they got inside’ (C16).

Participants frequently reported that the communication styles of healthcare staff did not cater to their level and that the information provided did not meet their needs, all of which impeded understanding of crucial information: ‘I could just tell the doctors were talking massive terminology. I would have to stop them… And I'd explain it to her and then she would nod’ (C16). ‘Reading medical documents, yeah 100% I had to ask one of the nurses or someone what it means. “Cause you know it's a different language”’ (P5). These examples demonstrate patients once again falling back on their safety net of advocacy and carers' support to receive crucial information.

The pattern of important messaging and health information not being tailored and appropriately delivered to First Nations people may result in participants not being adequately prepared and reassured for the care journey and missing the opportunity to be fully involved in care decisions. Specific communication strategies and models of information delivery offered by participants, e.g., ‘they need to break it down a bit… or do some drawings or… point your hands’ (P1), would likely facilitate better understanding and involvement for First Nations people impacted by HNC.

### 3.7 Gaps in Health System Support: A More Holistic Approach Required

Support systems provided through the healthcare pathway were noted during the yarns with First Nations participants, however, ongoing gaps and unmet needs remain. Despite practical support, such as free car parking in the radiation therapy building, one‐off grocery vouchers, and taxi fares to the hospital, being appreciated, most indicated ongoing financial strain: ‘Yeah, we had mortgage stress and bills coming out of our ears and I hadn't worked for that time’ (P3). ‘It was pretty hard but Grandma's son helped a lot, like he paid out all my bills because you know he's like you're looking after my mum.’ (C2). Additionally, most participants expressed an emotional toll from the HNC journey, which was overlooked: ‘So watching all the challenges and barriers. You know if he goes shopping, he just put it on the counter… ‘how much?’ and then he pays for it and that'll be it, there's no yarning… it's just watching a sad story’ (C6). ‘Having support or a little counselling session every so often…it was one thing after the other… it would have been helpful to have support or just the one‐on‐one with somebody to process it’ (C16). Furthermore, the significant impacts and burden on carers and family members given their essential roles tightening the weave of the safety net across the care pathway, were also largely not addressed by the health system or care staff: ‘I guess stressful, and I stopped my whole life. But I didn't work anymore, I gave my job up’ (C2). In addition, the travel funding scheme was unable to accommodate an additional escort as a support for both the patient as well as the primary carer, reflecting that the hospital system has yet to adapt to and recognise a kinship‐centred model of care for First Nations people.

This theme demonstrates that the health system and its existing supports fail to consider First Nations concepts of health and wellbeing; thereby, weakening the weave of the safety net, and a more considered and holistic approach is needed to improve the care experience.

## 4 Discussion

This is the first known study using Yarning and reflexive thematic analysis to understand the HNC care experiences for First Nations Australians, through the seven key themes developed from the data. Diversity in experiences was noted across most themes; however, strength and resilience being held up through community were consistently the most influential and beneficial aspects in the HNC care journey.

The integrative Strengths and protective factors theme highlighted family, spiritual connections, and kinship support as central to HNC care and wellbeing. This is widely reported in First Nations cancer care literature more generally, with families taking on and sharing similar support roles [26]. In addition, participants' personal qualities of resilience, self‐advocacy, and determination sustained them through the complexities of the health system and are in line with findings from Meiklejohn and colleagues [27]. However, unique to this cohort was the impact of HNC and its treatments on verbal communication. Whilst communication impairments associated with HNC are well‐documented [28], this study highlighted the resultant limitations on yarning, advocacy, and cultural connection for First Nations Australians. Considering this, participants emphasised how family helped to convey messages, anticipate care needs, provide a means of communication with the healthcare staff, and advocate for care, which in turn helped to promote the patient's ongoing self‐determination, even if their voice was temporarily or permanently lost. This was also reflected in recent findings by Checklin and colleagues [29] for a general HNC population, and underscores the need for health systems to adopt a family‐centred approach in order to strengthen the safety net that supports the patient.

Participants identified the need for more financial, practical, and psychological supports. This finding is consistent with previous work regarding unmet supportive care needs of First Nations patients with cancer more generally [30] and their carers [26]. Gaps in psychological support, as noted in the current study, are particularly detrimental to the HNC survivorship experience, given the likelihood of disfigurement or drastic physical changes [31], compromise of swallowing, communication and breathing functions [28], as well as high levels of reported distress by both patients and caregivers [31] associated with HNC and its treatment. Specifically in First Nations cultures, the treatment and impacts may conflict with the values to maintain the wholeness of the body [32], and so the psychological wellbeing of this cohort warrants additional attention.

First Nations participants in this cohort, the majority of which were from non‐metropolitan areas, shared experiences of additional stress and uncertainty relating to delays, disjointed and poor care coordination, as well as the necessity to travel for treatment, all weakening the weave of their safety net. These issues are consistent with previous literature findings from for the broader HNC population living in regional or remote settings [33, 34]. In addition, participants highlighted the need for increased information support, care coordination, local staff with specialist HNC knowledge, psychological support, as well as a desire for care closer to home, which all closely align with the work of Pradhan and colleagues [35] who examined survivorship needs for HNC patients from rural Australia. With connection to land, culture, and community being paramount to First Nations health and wellbeing [36], the inherent separation and disconnection during treatment may impact on identity, sense of self, and social and emotional wellbeing, necessitating additional culturally responsive, supportive care for First Nations Australians.

**FIGURE 3:**
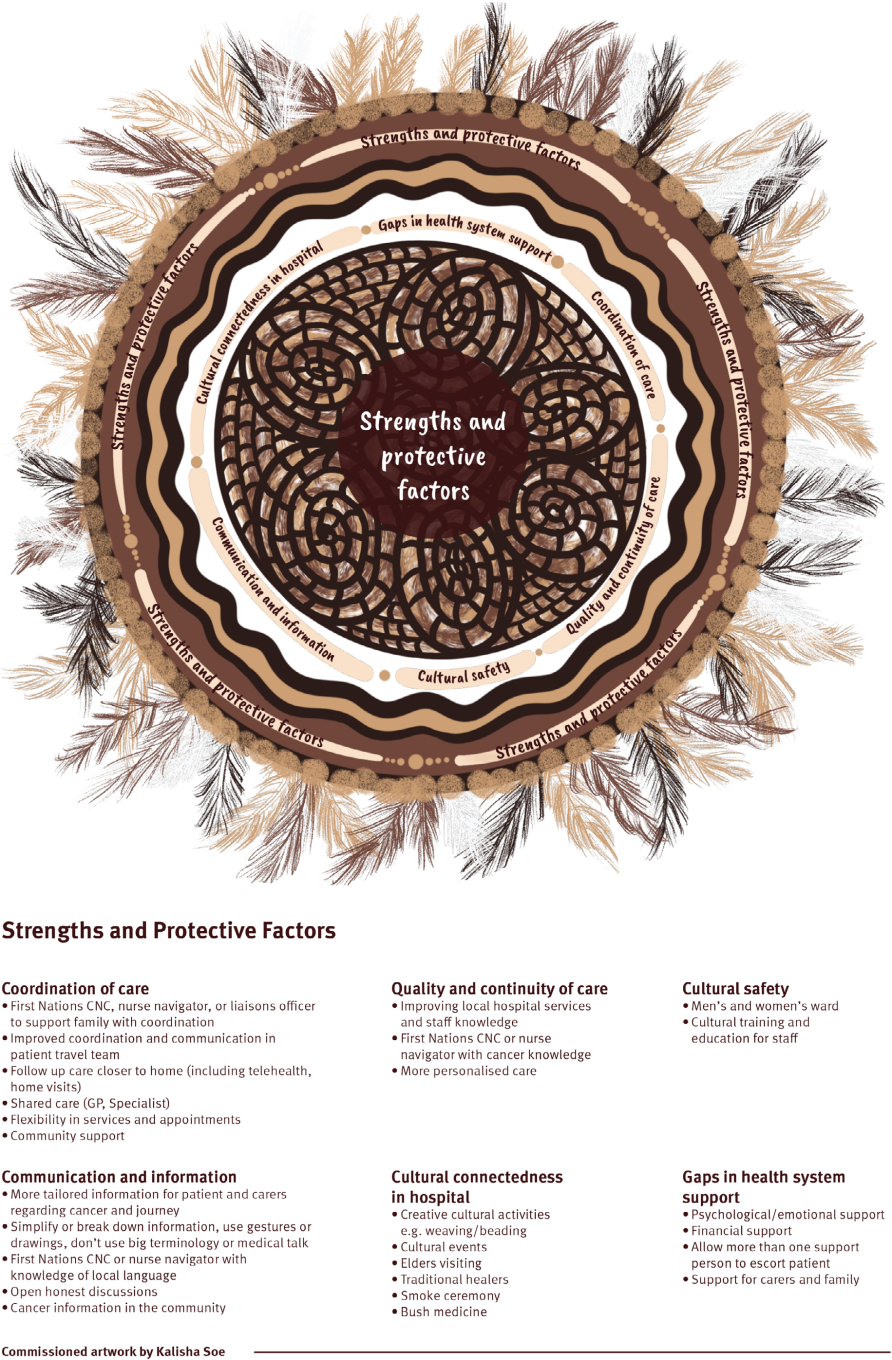
Learnings for clinical practice aligned with the interwoven themes.

Participants shared care experiences throughout the yarns, and in doing so offered practical suggestions on how the care pathway could be enhanced in the future. These learnings are collated under each theme, and if implemented represent a mechanism by which the safety net for First Nations patients and their families undergoing HNC care can be strengthened (Figure 3). Furthermore, the improvement initiatives may have broader applicability and benefit to a wider HNC cohort, particularly with regards to flexibility in care modalities, as well as increased financial and psychological support, and would better meet the guidance for follow up and survivorship care from the HNC OCP.

Participants in this current study repeatedly emphasised a need for a dedicated First Nations cancer coordinator role along the HNC pathway, both locally but also in the tertiary facility, to bridge the cultural and practical gaps along the journey. Effective care coordination, as reinforced in the OCP for Aboriginal and Torres Strait Islander people with cancer [14], has proven to be critical in helping to meet social, cultural, and logistical challenges in cancer care [37] and the wide‐ranging benefits of having a First Nations cancer coordinator role have been well‐documented in other cancer research more broadly [10, 13].

Whilst some participant‐generated recommendations from this study may necessitate large‐scale, system‐wide changes, some of the other practical suggestions for staff, such as cultural education and communication training, may prove easier to implement into clinical service delivery and make an immediate difference to strengthening the safety net of care. Healthcare staff and organisations recognising the barriers and power imbalances within the system, as well as consciously reflecting on their own biases and assumptions, are central to cultural safety [38], and cultural training for staff has been shown to effectively increase awareness of First Nations health issues and foster improved relationships and partnerships [39], setting the foundation for culturally safe care. Furthermore, successful communication can positively shape healthcare experiences and contribute to high quality care, and similar strategies have been shown to be effective in previous First Nations cancer care literature, such as selecting plain and simple language, using visual aids, involving family to help with discussions, and ensuring a safe and trusting relationship as a cornerstone for all interactions [12, 40].

### 4.1 Strengths and Limitations

This study captured a unique cohort that generously shared rich experiences to inform changes to policy and practice; however, the research team acknowledges that the small sample size may have varying applicability in statewide or nationwide health settings and rather the findings may form the initial focus point for change at a local level.

## 5 Conclusion

Yarning with HNC patients and carers enabled understanding of experiences through the current care pathway and generated recommendations to inform a future co‐design process to guide service improvement. Participants highlighted the importance of kinship‐centred models of care, streamlined coordination, timely and open communication, cultural training for staff, personalised care, as well as opportunities to maintain connection with culture and community in the yarns. These in turn would elevate the inherent strengths within family and kinship support that weave through and uphold the safety net through the challenges of the HNC journey.

## Author Contributions

**Joanne Tesiram:** conceptualization, investigation, writing – review and editing. **Stephanie Ng:** conceptualization, writing – original draft, formal analysis, data curation, project administration, writing – review and editing, software. **Kalisha Soe:** , investigation, writing – review and editing. **Lynette Koina:** formal analysis, writing – review and editing, conceptualization. **Gail Garvey:** conceptualization, formal analysis, supervision, writing – review and editing. **Rebecca Packer:** conceptualization, methodology, formal analysis, supervision, writing – review and editing. **Tamara Butler:** conceptualization, formal analysis, writing – review and editing, supervision, methodology. **Bena Brown:** conceptualization, methodology, formal analysis, supervision, project administration, funding acquisition, writing – review and editing. **Elizabeth C. Ward:** conceptualization, funding acquisition, supervision, formal analysis, writing – review and editing.

## Funding

S.N. was supported by a University of Queensland scholarship as part of the higher research degree program. The study was conducted as part of a larger funded body of work supported by a Project Grant from the Metro South Health Research Support Scheme, Queensland Health (Project Grant RSS 2023 008). T.B. was supported by National Health and Medical Research Council (NHMRC) Investigator Grant (GNT2008097), and GG was supported by a NHMRC Leadership Grant (GNT 2034453).

## Ethics Statement

This study was performed in line with the National Statement on Ethical Conduct in Human Research 2023 and in accordance with the Declaration of Helsinki—Ethical Principles for Medical Research Involving Human Participants. Specific approval for research with Aboriginal and Torres Strait Islander peoples was granted by the Metro South Health Human Research Ethics Committee (HREC/2023/QMS/92627). This study was conducted in accordance with NHMRC Ethical Guidelines for Research with Aboriginal and/or Torres Strait Islander people and the Australian Institute of Aboriginal and Torres Strait Islander Studies (AIATSIS) Code of Ethics for Aboriginal and Torres Strait Islander Research.

## Conflicts of Interest

The authors declare no conflicts of interest.

## Data Availability

Research data are not shared.
